# Plasma exchange-based artificial liver support for acute liver failure associated with early-onset intrahepatic cholestasis of pregnancy: a case report

**DOI:** 10.1186/s12884-026-09844-1

**Published:** 2026-08-19

**Authors:** Yannan Zhu, Zuoxin Qin, Haiyan Tang, Bangmin Li

**Affiliations:** 1 Department of Obstetrics and Gynecology, The 924th Hospital of the Joint Logistics Support Force of the Chinese People’s Liberation Army, Guilin, China; 2 Unit 75180, The Chinese People’s Liberation Army, Guilin, China

**Keywords:** Intrahepatic cholestasis of pregnancy, Acute liver failure, Artificial liver support system, Plasma exchange, Case report

## Abstract

**Background:**

Intrahepatic cholestasis of pregnancy (ICP) is typically benign, but progression to acute liver failure (ALF) is a rare, life-threatening complication. We report a case of ALF associated with early-onset ICP managed successfully with plasma exchange-based artificial liver support system (ALSS).

**Case presentation:**

A 29-year-old Chinese woman (G2P1) presented with abnormal liver function at 20 weeks of gestation, characterized by markedly elevated aminotransferases but only mildly increased total bile acid (TBA, 12.4 µmol/L). Despite supportive care, she developed progressive cholestasis, hyperbilirubinemia, coagulopathy, and mild encephalopathy. Pregnancy was terminated at 23 weeks due to disease progression. Post-termination, TBA surged to 360.9 µmol/L, indicating persistent severe cholestasis. Given the poor response to conventional therapy, sequential ALSS combining Double Plasma Molecular Adsorption System (DPMAS) and plasma exchange was initiated. The patient exhibited rapid biochemical improvement and recovered fully without liver transplantation.

**Conclusions:**

This case illustrates a rare clinical presentation of acute liver failure associated with early-onset intrahepatic cholestasis of pregnancy in the setting of multiple potential contributing factors. Plasma exchange-based artificial liver support may provide temporary extracorporeal support for selected patients with progressive pregnancy-associated hepatic dysfunction. Further studies are required to clarify the optimal timing, indications, and clinical benefits of this therapy.

## Introduction

ICP is the most common pregnancy-specific liver disorder and is characterized by impaired hepatobiliary transport and elevated maternal TBA levels [1, 2]. Although most patients recover after delivery, severe ICP, particularly with markedly elevated bile acid concentrations, is associated with increased risks of adverse fetal outcomes, including preterm birth, meconium-stained amniotic fluid, and stillbirth [3, 4].

Early-onset ICP, defined as disease onset before 28 weeks of gestation, is relatively uncommon and is often associated with more severe biochemical abnormalities and higher risks of adverse pregnancy outcomes [4, 5]. Its pathogenesis is considered multifactorial, involving pregnancy-related hormonal changes, genetic susceptibility, and inflammatory or environmental factors. Variants in hepatobiliary transporter genes, including *ABCB4* (MDR3), *ABCB11* (BSEP), and *ATP8B1* (FIC1), have been reported in patients with severe or recurrent ICP, suggesting a potential contribution of genetic factors to disease severity [6–8].

Although ICP commonly causes cholestatic liver dysfunction, progression to ALF is extremely rare [9]. When patients with ICP develop progressive liver injury, coagulation abnormalities, or neurological manifestations, alternative or additional causes, such as drug-induced liver injury, viral infection, autoimmune hepatitis, and other pregnancy-associated liver diseases, should be carefully evaluated [10]. Pregnancy-associated ALF is a critical condition requiring prompt multidisciplinary management. In addition to timely termination of pregnancy when indicated, extracorporeal liver support therapies, including plasma exchange-based artificial liver support system (ALSS), may provide temporary hepatic support in selected patients with severe liver dysfunction [11].

Herein, we report a rare case of ALF associated with early-onset ICP in a patient with progressive cholestasis, coagulation abnormalities, and mild neurological manifestations. The patient was successfully managed with multidisciplinary treatment combined with plasma exchange-based artificial liver support. This case highlights the diagnostic challenges of severe pregnancy-associated liver dysfunction and provides clinical insights into the potential role of ALSS in selected patients.

## Case presentation

A 29-year-old Chinese woman (G2P1) was admitted at 20 + weeks of gestation on February 3, 2025, because of abnormal liver function detected during routine prenatal examination. She had no nausea, vomiting, abdominal pain, jaundice, or pruritus, and physical examination was unremarkable.

Approximately 20 days before admission, she had influenza A infection with a maximum temperature of 39 °C and received oseltamivir, acetaminophen, and symptomatic treatment. Liver and renal function tests at that time were normal.

Initial laboratory investigations showed markedly elevated aminotransferases (ALT 471 U/L; AST 458 U/L), mildly elevated TBA (12.4 µmol/L), normal bilirubin levels, and preserved coagulation function. Abdominal ultrasonography demonstrated slightly coarse hepatic echotexture without biliary obstruction.

The patient was initially diagnosed with acute liver injury during pregnancy with suspected early-onset ICP. Because of the atypical presentation, alternative etiologies were systematically evaluated. Serological tests for hepatitis A, B, C, and E viruses were negative. Antinuclear antibody was positive, whereas autoimmune hepatitis could not be confirmed because liver biopsy, serum IgG, anti-smooth muscle antibody, and anti-liver kidney microsomal antibody testing were unavailable. Drug-induced liver injury (DILI) was also considered because of recent exposure to oseltamivir, acetaminophen, and subsequently ceftriaxone.

Despite treatment with ursodeoxycholic acid hepatoprotective agents, liver injury progressed rapidly. By February 6, serum aminotransferases exceeded 1,100 U/L, accompanied by increasing TBA levels, supporting the diagnosis of early-onset ICP. Between February 7 and 10, the patient developed recurrent fever and respiratory symptoms. Adenovirus DNA was detected, and inflammatory markers were mildly elevated. Following infectious disease consultation, ceftriaxone was initiated for suspected bacterial co-infection.

On February 12, progressive jaundice and dark urine developed, accompanied by a marked increase in TBA and persistent liver dysfunction. Infection-related liver injury and DILI were considered, and methylprednisolone therapy was initiated because of suspected immune-mediated drug-related liver injury. Three days later, the patient developed a generalized pruritic erythematous rash. Dermatological evaluation favored a morbilliform drug eruption, and ceftriaxone was discontinued. Intravenous immunoglobulin and supportive therapy were administered, resulting in gradual resolution of the rash; however, severe cholestasis persisted.

The patient’s condition subsequently deteriorated with progressive hepatic dysfunction, coagulation abnormalities, and mild neurological manifestations, including excessive somnolence and altered mental status (Table 1). After multidisciplinary discussion involving specialists in obstetrics, hepatology, infectious diseases, intensive care, and anesthesiology, acute liver failure associated with early-onset ICP was considered the most likely diagnosis. Given the progressive maternal deterioration, labor induction was performed at 23 weeks of gestation on February 19, 2025.

**Table 1:** Dynamic changes of laboratory parameters during clinical course

Indicators	Reference Range	Admission (Feb 3, 2025)	Peak Value During Disease Progression	After ALSS Treatment (Mar 5, 2025)
TBA (µmol/L)	< 10	12.4	360.9 (Feb 22)	4.0
TBIL (µmol/L)	5–21	9.0	242.3 (Feb 18)	28.5
ALT (U/L)	< 40	471	1160 (Feb 6)	40
AST (U/L)	< 35	458	1203 (Feb 6)	38
PT (s)	11–14.5	12.0	17.6 (Feb 18)	12.8
INR	0.8–1.2	1.0	1.54 (Feb 18)	1.05
APTT (s)	25–35	30.1	100.3 (Feb 18)	29.9
TT (s)	14–21	18.7	92.8 (Feb 18)	20.2
Fib (g/L)	2.0–4.0	3.0	1.44 (Feb 18)	3.1

Peak values of coagulation parameters and bilirubin preceded the peak of TBA, reflecting the sequential progression from hepatocellular injury to severe cholestasis and synthetic dysfunction

*ALSS* Artificial liver support system, *TBA* Total bile acid, *TBIL* Total bilirubin, *ALT* Alanine aminotransferase, *AST* Aspartate aminotransferase, *PT* Prothrombin time, *INR* International normalized ratio, *APTT* Activated partial thromboplastin time, *TT* Thrombin time, *Fib* Fibrinogen

Following pregnancy termination, aminotransferase levels gradually declined; however, severe cholestasis persisted, with serum TBA continuing to increase despite improvement in hepatocellular injury. Because of persistent cholestasis, coagulation abnormalities, and ongoing risk of hepatic deterioration, plasma exchange-based ALSS therapy was initiated on February 22, 2025.

The patient underwent sequential Double Plasma Molecular Adsorption System (DPMAS) followed by plasma exchange. DPMAS was performed for 3 h (14:40 − 17:40) with a plasma adsorption rate of 1.7 L/h and a total processed plasma volume of 5.1 L. This was immediately followed by plasma exchange for 90 min (17:50 − 19:20), with an exchange rate of 1.0 L/h and a total plasma exchange volume of 1.5 L. The procedure was well tolerated without allergic reactions, hemodynamic instability, or bleeding complications.

Following ALSS treatment, serum bilirubin, bile acid levels, liver enzymes, and coagulation parameters gradually improved. The patient continued supportive care and was discharged in stable condition on March 7, 2025.

At one-year follow-up, liver function had completely normalized (ALT 19 U/L, AST 17 U/L, TBA 2.7 µmol/L, and total bilirubin 15.5 µmol/L). She remained asymptomatic and had resumed normal daily activities. Preconception counseling was provided regarding the risk of ICP recurrence and the need for early monitoring of liver function and serum bile acid concentrations in future pregnancies.

## Discussion

### Clinical characteristics and diagnostic challenges

ICP is characterized by impaired hepatobiliary transport and elevated maternal TBA concentrations [1, 2]. Although ICP is associated with significant fetal risks, maternal prognosis is generally favorable, and progression to ALF is extremely rare [3]. In this case, the patient developed early-onset ICP followed by rapidly progressive hepatic dysfunction, coagulation abnormalities, hyperbilirubinemia, and mild neurological manifestations, representing an unusually severe phenotype of pregnancy-associated liver disease.

The diagnosis and etiology of ALF were challenging because the initial presentation was atypical for severe ICP. The patient initially showed markedly elevated aminotransferases with only mildly increased TBA levels (12.4µmol/L). Therefore, alternative causes of liver injury, including DILI, infection-associated liver injury, autoimmune hepatitis, and other pregnancy-related liver disorders, were carefully considered [4, 5].

The patient had recent influenza A infection and exposure to multiple medications, including oseltamivir, acetaminophen, and ceftriaxone. The development of a morbilliform drug eruption during hospitalization further suggested a possible drug-related contribution [6]. Because comprehensive autoimmune evaluation and genetic testing were unavailable, a multifactorial contribution to liver injury cannot be excluded.

Therefore, this case should be interpreted as ALF associated with early-onset ICP in the setting of multiple potential contributing factors rather than as definitive ALF caused solely by ICP [3, 7].

### Potential mechanisms underlying severe disease progression

The mechanisms underlying severe maternal hepatic dysfunction in ICP remain incompletely understood. Current evidence suggests that ICP results from interactions among genetic susceptibility, hormonal changes, and environmental factors.

Early-onset ICP may represent a more severe clinical phenotype with a higher likelihood of underlying hepatobiliary transport abnormalities. Variants in bile acid transporter genes, including *ABCB4* (MDR3), *ABCB11* (BSEP), and *ATP8B1* (FIC1), have been reported in patients with severe or recurrent ICP [9–11]. These abnormalities may impair bile acid secretion and increase susceptibility to pregnancy-related hormonal stress.

Although genetic testing was not available in this patient, a potential genetic predisposition cannot be excluded. In addition, a multi-hit mechanism may have contributed to disease progression [12]. Pregnancy-related hormonal changes, early-onset cholestatic susceptibility, and additional stressors such as infection and medication exposure may have collectively impaired hepatic adaptation, leading to severe hepatic dysfunction despite relatively low initial TBA levels [13].

### Persistent cholestasis after pregnancy termination

Termination of pregnancy is considered the definitive treatment for ICP because removal of the hormonal stimulus usually leads to improvement of cholestasis. However, recovery may be delayed in patients with severe hepatic dysfunction or underlying transport defects [14].

In this case, aminotransferase levels decreased after pregnancy termination, indicating partial recovery of hepatocellular injury. However, serum TBA continued to increase, reaching 360.9 µmol/L after termination. This discrepancy between improving aminotransferases and persistent worsening cholestasis suggests that bile acid transport dysfunction remained active despite improvement in hepatocellular damage [15].

Persistent severe cholestasis may contribute to ongoing systemic toxicity and delayed hepatic recovery, indicating that additional supportive therapies may be considered in selected patients with progressive disease.

### Role of plasma exchange-based artificial liver support

ALSS have been increasingly used as temporary extracorporeal therapies for patients with severe hepatic dysfunction who may recover with supportive treatment [16]. In this patient, plasma exchange-based ALSS was initiated because of persistent severe cholestasis, coagulation abnormalities, and insufficient improvement after pregnancy termination and conventional management. Following DPMAS combined with plasma exchange, liver biochemical parameters and coagulation function progressively improved, and liver transplantation was avoided [11].

However, the effectiveness of ALSS in ICP-associated ALF remains uncertain because current evidence is limited to case reports and small observational studies [17]. Therefore, ALSS should be considered a temporary supportive therapy for carefully selected patients rather than an established alternative to transplantation.

### Limitations

This report has several limitations. First, as a single case report, it cannot establish a causal relationship between ICP and ALF [18]. Second, genetic testing for ICP-associated transporter gene variants was not performed; therefore, a potential genetic predisposition could not be assessed. Third, although clinical features supported the diagnosis of ALF, prognostic scoring systems such as the Model for End-Stage Liver Disease (MELD) score and King’s College Criteria were not prospectively calculated, which may limit the generalizability of our findings regarding disease severity assessment [19, 20]. Finally, the relative contributions of pregnancy-related cholestasis, infection, and medication exposure to hepatic injury cannot be completely separated.

## Conclusion

In conclusion, ALF associated with early-onset ICP represents a rare but life-threatening condition requiring comprehensive differential diagnosis and multidisciplinary management. In this case, severe cholestasis and hepatic dysfunction persisted despite pregnancy termination. Plasma exchange-based artificial liver support served as an effective temporary bridge therapy, facilitating recovery and averting the need for liver transplantation. While high-level evidence remains limited, our findings suggest that ALSS may be a viable salvage option in selected patients with progressive hepatic dysfunction and delayed biochemical improvement following delivery [17].

## Data Availability

No datasets were generated or analysed during the current study.
